# Sugammadex facilitates early recovery after surgery even in the absence of neuromuscular monitoring in patients undergoing laryngeal microsurgery: a single-center retrospective study

**DOI:** 10.1186/s12871-016-0221-2

**Published:** 2016-08-02

**Authors:** Eui-Seok Park, Byung Gun Lim, Won-Joon Lee, Il Ok Lee

**Affiliations:** Department of Anaesthesiology and Pain Medicine, Korea University Guro Hospital, Korea University College of Medicine, 148, Gurodong-ro, Guro-gu, Seoul, 08308 Republic of Korea

**Keywords:** Enhanced recovery after surgery (ERAS), Microsurgery, Neuromuscular blocking agents, Neuromuscular monitoring, Pyridostigmine bromide, Rocuronium, Sugammadex

## Abstract

**Background:**

In many countries, routine clinical anaesthesia does not always involve neuromuscular monitoring. In these clinical settings, the efficacy and safety of sugammadex use has not yet been confirmed. We investigated the efficacy and safety of sugammadex in the absence of neuromuscular monitoring.

**Methods:**

One hundred and forty patients who underwent laryngeal microsurgery with the use of rocuronium as a neuromuscular blocking agent, without the use of a neuromuscular monitoring device, were retrospectively investigated. The patients were randomly chosen among all the patients who met the inclusion criteria at a tertiary university hospital between July 2013 and February 2015 and were allocated to group S (sugammadex group) or group P (pyridostigmine group) according to the neuromuscular reversal agent administered. Five patients were excluded from analysis and 135 patients completed the study. Primary outcome was extubation time. Secondary outcomes were anaesthesia time, the correlation between anaesthesia time and extubation time, the total amount of rocuronium, and postoperative adverse events in the post-anaesthesia care unit (PACU).

**Results:**

Extubation time was significantly shorter in group S (6.3 ± 3.9 min) than in group P (9.0 ± 5.4 min). Anaesthesia time was also significantly shorter in group S (30.7 ± 10.3 min) than in group P (35.8 ± 12.6 min). In the patients with an anaesthesia time of 30 min or less, there was a positive correlation between anaesthesia time and extubation time in group P (*r* = 0.453), but there was no significant relationship in group S. The total amount of rocuronium used was higher in group S (0.62 ± 0.11 mg kg^−1^) than in group P (0.38 ± 0.14 mg kg^−1^). Postoperative adverse events in the PACU were comparable between the groups, except for tachycardia events: the incidence of tachycardia was significantly lower in group S (8.0 %) than in group P (17.3 %).

**Conclusions:**

Sugammadex could shorten anaesthesia and extubation times as well as recovery time in the PACU and reduce postoperative hemodynamic complications in a clinical setting in the absence of neuromuscular monitoring. This may enhance the patients’ recovery in the operating room and PACU while improving the postoperative condition of patients.

**Trial registration:**

The trial was registered in the UMIN clinical trials registry (www.umin.ac.jp/ctr/index/htm; unique trial number: UMIN000016602; registration number: R000019266; principal investigator’s name: Byung Gun Lim; date of registration: February 22, 2015).

**Electronic supplementary material:**

The online version of this article (doi:10.1186/s12871-016-0221-2) contains supplementary material, which is available to authorized users.

## Background

The recommended dose of sugammadex ranges from 2 to 16 mg kg ^−1^ and is based on an objective assessment of the depth of the neuromuscular block [1]. Such assessments require the aid of appropriate neuromuscular monitoring devices. However, in Korea, routine clinical anaesthesia does not usually involve neuromuscular monitoring, because of lack of understanding of the need for monitoring, or because anaesthesiologists are not yet accustomed to such monitoring, or find it an inconvenience. This is also not uncommon elsewhere in the world [2–5]. In such clinical setting, the efficacy and safety of sugammadex use has not yet been clearly confirmed.

Sugammadex has been demonstrated to shorten the time to reach a train-of-four ratio of 0.9 as well as the extubation time, resulting in improvement of operating room turnover in clinical anaesthesia settings, including neuromuscular monitoring [6, 7]. Therefore, sugammadex may be a useful factor for the enhanced recovery after surgery (ERAS) protocol [8]. However, this viewpoint has not yet been reported in a clinical setting in the absence of neuromuscular monitoring.

Sugammadex may be recommended for the reversal of neuromuscular block in surgery that requires deep blockade to facilitate surgical procedures or that requires very short surgical time [6]. From this point of view, laryngeal microsurgery meets the indications for sugammadex use. Therefore, we frequently use this agent for the reversal of neuromuscular block in laryngeal microsurgery, although we do not implement neuromuscular monitoring, including train-of-four monitoring, for the process.

We therefore aimed to investigate whether sugammadex shortens anaesthesia time, extubation time, and recovery time in the postoperative period, and to establish whether it reduces postoperative adverse events, as compared with pyridostigmine, one of the reversal agents most commonly used in our hospital. In our hospital, the administration of rocuronium and its reversal agent is not guided by neuromuscular monitoring in patients undergoing laryngeal microsurgery. We hypothesized that sugammadex could shorten anaesthesia and extubation times, as well as the recovery time during the postoperative period, and that it can reduce postoperative haemodynamic complications in patients undergoing laryngeal microsurgery, even in the absence of neuromuscular monitoring

## Methods

Ethical approval for this study was provided by the Korea University Guro Hospital Institutional Review Board, Seoul, Republic of Korea on 31 January 2015 (approval number: KUGH15105-002). The requirement for written informed consent from the patients was waived by the institutional review board because the design of this study was a retrospective study. The trial was registered in the UMIN clinical trials registry (unique trial number: UMIN000016602; registration number: R000019266; principal investigator’s name: Byung Gun Lim; date of registration: 22 February 2015).

### Patient population, study protocol and data collection

One hundred and forty patients who underwent elective laryngeal microsurgery with the use of rocuronium as the intraoperative neuromuscular blocking agent, without the use of an intraoperative neuromuscular monitoring device, at the Korea University Guro Hospital from July 2013 to February 2015 were retrospectively investigated through the electronic medical record system. Patients were all American Society of Anaesthesiologists (ASA) physical status I or II and aged 19–75 years. Since the proper sample size calculated from the result of a pilot study was 70 for each group, among all the patients who met the inclusion criteria since the introduction of sugammadex use at our hospital up to the initiation of this study, 70 patients from those to whom sugammadex was administered and 70 patients from those to whom pyridostigmine was administered were chosen randomly using a random integer generator program. Five patients with unusually long operation time, due to difficulties in performing either endotracheal intubation or the surgery, were excluded, and a total of 135 patients were finally allocated to group S (sugammadex group; *n* = 68) or group P (pyridostigmine group; *n* = 67) according to the neuromuscular reversal agent that had been administered. The dose of the neuromuscular reversal agent administered at each group was as follows: The dose of sugammadex was 2.3 ± 0.5 mg kg^−1^ in group S and the dose of pyridostigmine was 10 mg in all patients in group P. All data, including patients’ characteristics, and primary and secondary outcomes, were collected from the electronic medical record system by an investigator unaware of the purpose of the study.

### Study endpoints

The primary outcome was extubation time which was measured as the time interval from the end of surgery to extubation. Secondary outcomes were anaesthesia time, the correlation between anaesthesia time (the time interval from intubation to extubation) and extubation time in patients with an anaesthesia time of 30 min or less, the total amount of rocuronium and reversal agents used, the number of patients in whom additional rocuronium, beside the initial injection, was required during surgery, the interval between the end of surgery and the administration of reversal agents, the interval between the administration of the last dose of rocuronium and the administration of reversal agents, the time to reach a Ramsay sedation score of 2 and the recovery time in the post-anaesthesia care unit (PACU) (the time to reach a modified Aldrete score of 10 from entering the PACU), and postoperative adverse events in PACU, including respiratory and haemodynamic complications, postoperative residual weakness, postoperative nausea and vomiting (PONV) or pain (specifically, desaturation [SpO_2_ < 93 %], laryngospasm [upper airway obstruction], tachypnea or apnea, tachycardia or bradycardia, hypertension or hypotension, severe PONV, or pain requiring anti-emetics or analgesics). Tachycardia, bradycardia, hypertension, and hypotension were considered positive if the values exceeded ± 20 % of the pre-induction vital signs.

### Sample size calculation

The primary endpoint in this study was extubation time. The sample size calculation was based on the results of extubation time from a pilot study involving 10 cases in each group. In the pilot study, extubation times (mean ± standard deviation) were 6.7 ± 3.9 min in group S and 9.5 ± 6.8 min in group P. Therefore, the effect size of a 2-group study was 0.5. On the assumption that the allocation ratio was 1, and with a sample size of 63 for each group, a power of 0.8 at a level of significance of 0.05 would be achieved (calculated by a two-sided Student’s *t*-test). Considering a 10 % dropout rate, the sample size for final enrolment was 70 in each group.

### Statistical analysis

Statistical analyses were performed using the Statistical Package for Social Sciences, version 12.0 (SPSS, Chicago, IL, USA). Categorical data, including sex, ASA class, operation type, the number of patients given additional rocuronium, and the incidence of postoperative adverse events between the groups were compared using a chi-squared test or Fisher’s exact test. Other parametric data, including further demographic data, such as age, height etc., anaesthesia time, extubation time, the total amount of rocuronium injected, the time interval between the end of surgery and the injection of reversal agents, the time interval between the administration of the last dose of rocuronium and the administration of reversal agents, the time to reach a Ramsay sedation score of 2, and the recovery time in the PACU were compared using a two-tailed Student’s *t*-test (normally distributed data) or the Mann–Whitney *U*-test (non-normally distributed data). The relation between anaesthesia time and extubation time in each group was analyzed with Spearman Rank Order Correlation analysis. A *P*-value < 0.05 was considered statistically significant. We performed Bonferroni correction to reduce the chances of obtaining false-positive results (type I errors) when multiple pair wise tests are performed on a single set of data in the statistical analysis of secondary outcomes regarding time intervals which were partly overlapping (the interval between the administration of the last dose of rocuronium and the administration of reversal agents vs. the interval between the end of surgery and the administration of reversal agents, and the time to reach a Ramsay sedation score of 2 vs. the recovery time in the PACU). In the analysis of these outcomes, a *P*-value < 0.025 was considered statistically significant.

## Results

**Fig. 1:**
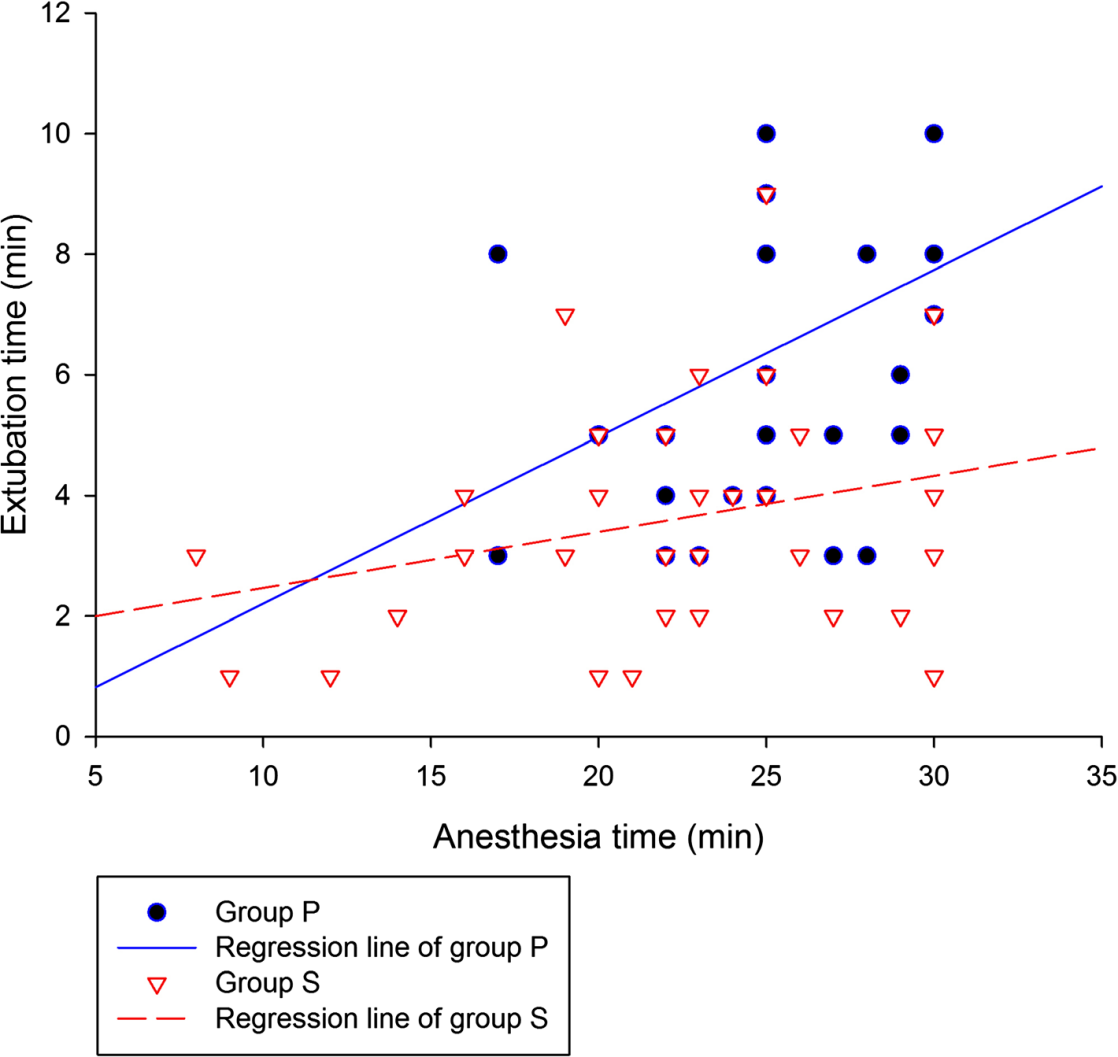
The correlation between anaesthesia time and extubation time. In patients with an anaesthesia time of 30 min or less (32 and 34 patients in groups P and S, respectively), there was a significant positive correlation between anaesthesia time and extubation time in group P (*r* = 0.453; *P* = 0.01), but no significant correlation was seen in group S (*r* = 0.271; *P* = 0.12). Group P: patients who received pyridostigmine as a reversal agent. Group S: patients who received sugammadex as a reversal agent

A total of 135 patients were evaluated, with 68 patients included in group S and 67 patients in group P. The demographic and clinical data were not significantly different between the groups (Table 1). Extubation time was significantly (*P* = 0.002) shorter in group S (6.3 ± 3.9 min) than in group P (9.0 ± 5.4 min). Anaesthesia time was also significantly (*P* = 0.043) shorter in group S (30.7 ± 10.3 min) than in group P (35.8 ± 12.6 min). In the patients with an anaesthesia time of 30 min or less (32 and 34 patients in groups P and S, respectively), there was a positive correlation between anaesthesia time and extubation time in group P (*r* = 0.453; *P* = 0.01), but no significant relationship in group S (*r* = 0.271; *P* = 0.12; Fig. 1). The total amount of rocuronium used was higher in group S (0.62 ± 0.11 mg kg^−1^) than in group P (0.38 ± 0.14 mg kg^−1^; *P* ≤ 0.001). Patients in group P needed additional rocuronium during the surgery in more cases than did those in group S (14 vs. 2, respectively; *P* = 0.003). Among secondary outcomes regarding time intervals, the time to reach a Ramsay sedation score of 2, and the recovery time in the PACU were significantly shorter in group S than in group P (since every patient reached a modified Aldrete score of 10 upon reaching a Ramsay sedation score of 2, the times to reach these two criteria were the same). The interval between the end of surgery and the administration of reversal agents, and the interval between the administration of the last dose of rocuronium and the administration of reversal agents were comparable between the groups (Table 2).

**Table 1:** Demographic and clinical data of patients

Variables	Group S (*n* = 68)	Group P (*n* = 67)
Age (years)	51.8 ± 12.9	52.9 ± 12.0
Sex (M/F)	45/23	46/21
Height (cm)	165.3 ± 8.2	165.3 ± 8.8
Weight (kg)	66.6 ± 12.2	66.9 ± 12.1
ASA class (I/II)	29/39	23/44
Operation type (Biopsy/Resection)	10/58	10/57
Induction propofol/thiopental dose (mg kg^−1^)	1.9 ± 0.2 / 4.7 ± 0.3	1.84 ± 0.26 / 4.77 ± 0.48
Operation time (min)	9.6 ± 6.0	10.0 ± 7.0
Insp. SEVO/DES (vol %) at the end of surgery	2.15 ± 0.55 / 6.16 ± 0.68	2.35 ± 0.6 / 6.04 ± 1.06

The patient data are presented as mean ± SD or number of patients. There was no statistically significant difference between the groups for any of the variables. Group S: patients who received sugammadex as a reversal agent. Group P: patients who received pyridostigmine as a reversal agent

*M* male, *F* female, *ASA* American Society of Anaesthesiologists, *Insp.* Inspiratory, *SEVO* Sevoflurane, *DES* Desflurane

**Table 2:** Secondary outcomes regarding time intervals measured in the operating room and PACU

Variables	Group S (*n* = 68)	Group P (*n* = 67)	*P*-value
Reversal agent injection interval 1^a^ (min)	4.9 ± 3.2	6.8 ± 4.8	0.026
Reversal agent injection interval 2^b^ (min)	22.3 ± 8.3	25.7 ± 10.4	0.084
Recovery time in PACU^c^ (min)	3.1 ± 5.3*	8.1 ± 9.0	≤0.001
Time to reach Ramsay sedation score 2 in PACU (min)	3.1 ± 5.3*	8.1 ± 9.0	≤0.001

The patient data are presented as mean ± SD. Group S: patients who received sugammadex as a reversal agent. Group P: patients who received pyridostigmine as a reversal agent

*PACU* post-anaesthesia care unit

*P < 0.025 vs. group P (Bonferroni correction)

^a^the interval between the end of surgery and the administration of reversal agents

^b^the interval between the administration of the last dose of rocuronium and the administration of reversal agents

^c^the time to reach modified Aldrete score 10

Postoperative adverse events in the PACU were comparable between the groups, except for tachycardia events (Table 3). The incidence of tachycardia was significantly (*P* ≤ 0.001) lower in group S (8.0 %) than in group P (17.3 %). There was one case with PONV who required rescue antiemetics in group S and none in group P; this was not statistically significant. The requirement for postoperative analgesics was similar between the two groups (five cases in group S, four cases in group P). There was no desaturation (SpO_2_ < 93 %) event in group S, but there was one such case in group P; this case did not fully recover until 28 min after the initial rocuronium injection of 0.3 mg kg^−1^, which was 10 min after the injection of pyridostigmine (10 mg) in the operating room. The patient was moved to the PACU, intubated, and showed an SpO_2_ < 95 % even with a 6 L min^−1^ of O_2_ supply connected to the endotracheal tube. The patient required a further 20 min in the PACU to recover to the point where extubation was possible. He was suspected to have postoperative residual neuromuscular blockade. There was no laryngospasm or apnea event in either group.

**Table 3:** Postoperative adverse events in PACU

Variables	Group S (*n* = 68)	Group P (*n* = 67)	*P*-value
PONV required to use rescue antiemetics (n)	1	None	0.994
Hypertension events (%)	3.0	2.9	0.977
Hypotension events (%)	4.1	6.4	0.17
Tachycardia events (%)	8.0*	17.3	≤0.001
Bradycardia events (%)	7.8	10.7	0.156
Incomplete reversal case with hypoxia (n)	None	1	0.994

The patient data are presented as number of patients (n) or incidence of haemodynamic events (%). Group S: patients who received sugammadex as a reversal agent. Group P: patients who received pyridostigmine as a reversal agent

*PACU* post-anaesthesia care unit, *PONV* Postoperative nausea and vomiting

*P < 0.05 vs. group P

## Discussion

Since its introduction, sugammadex showed marked efficacy and safety, ringing in a new era of patient safety in anaesthesiology [7]. However, since its dosing regimen necessitates the use of neuromuscular monitoring, the use of this agent may be limited in certain situations, particularly where such monitoring devices are not routinely used. Several surveys have shown that this is not uncommon, worldwide [2–5]. Hence, we here performed a retrospective study on the efficacy and safety of using sugammadex in the absence of guidance provided by neuromuscular monitoring. We considered that this study could show whether sugammadex reduces anaesthesia and extubation times, as well as recovery time in the PACU, facilitating early recovery after surgery, even in the absence of neuromuscular monitoring, in a routine clinical anaesthesia setting, without any intervention.

The results of the present study showed that sugammadex shortened anaesthesia time, extubation time, and recovery time, without increasing the incidence of postoperative adverse events. The effect of sugammadex in shortening anaesthesia time, extubation time, and recovery time has been clearly demonstrated by many previous studies performed around the globe in settings using appropriate neuromuscular monitoring. The result of the present study further showed that the efficacy of sugammadex remains firm, even in the absence of proper neuromuscular monitoring [9–11].

Sugammadex not only enabled shorter extubation time even with larger dose of rocuronium but also, though not given at the dose guided by proper neuromuscular monitoring, reversed the neuromuscular block within relatively constant time in our study. In group P, the extubation time was longer and the variation in this time was larger. There was a positive correlation between anaesthesia time and extubation time in group P, but not in group S, which indicated that extubation time in group P functioned as a key component influencing the anaesthesia time, whereas the relatively constant extubation time in group S was not a main determinant of anaesthesia time, even in the absence of neuromuscular monitoring.

The shortened anaesthesia time, extubation time, and recovery time in group S may allow faster operating room turnover, and this can allow better patient prognosis in terms of overall safety and recovery. ERAS has recently gained increasing attention. Enhanced recovery protocols for perioperative care have been proven to reduce complications after surgery, improve overall outcomes, and shorten the length of hospital stay, thus saving on resources [12]. Thus, guidelines for specific fields are being formulated and are being published throughout the world [13–16]. According to the ERAS protocol developed by the ERAS society, using short-acting anesthetic agents is one of the elements comprising the intraoperative component of the protocol [17]. Due to its ability to reverse rocuronium-induced neuromuscular blockade quickly, sugammadex combined with rocuronium can work as a short-acting agent, even capable of substituting for succinylcholine [18, 19]. Hence, sugammadex combined with rocuronium can reduce anaesthesia time, recovery time, and length of hospital stay, making it recommendable as a short-acting anesthetic agent in the ERAS protocol [20, 21]. Based on the results of the present study, we strongly recommend use of sugammadex combined with rocuronium even in a clinical anaesthesia setting without neuromuscular monitoring as a new element in the ERAS protocol.

As for the adverse events, although not statistically significant, there was one case of incomplete reversal in group P, which can cause detrimental complications, whereas no such case was found in group S [22, 23]. As is known from several previous studies and surveys, anaesthesiologists in our hospital tend also to use larger doses of rocuronium to achieve a theoretical deeper blockade when sugammadex is planned for use at the end of surgery; although obtaining a deeper block in group S was not proven by neuromuscular monitoring, this trend is clearly reflected in our study, as the total amount of rocuronium used in group S was significantly larger than that used in group P [24]. This trend for obtaining a theoretical deeper block, associated with sugammadex in group S, highlights another benefit of sugammadex, in that there were only two cases that needed additional rocuronium during the surgery in group S, whereas group P required this in 14 cases. It can be assumed that additional rocuronium was needed because of incomplete or insufficient neuromuscular block, in order to achieve optimal surgical conditions, resulting in the possibility of elongated anaesthesia time, a shorter time between the last injection of rocuronium and the injection of the reversal agent, and a consequently higher risk of incomplete reversal of neuromuscular block or recurarization [8, 25]. Postoperative haemodynamic values were found to be better in group S, and the incidence of tachycardia was less. Glycopyrrolate injected along with pyridostigmine in order to block its cholinergic adverse effects may explain the significantly higher incidence of tachycardia in group P [26].

The limitation of this study is that it was a retrospective study. We plan a randomized controlled trial on this subject, to further assess its implications for the ERAS protocol and the possible use of sugammadex in combination with rocuronium in the ERAS protocol, in the near future.

## Conclusions

Sugammadex could shorten anaesthesia and extubation times, as well as the recovery time in the PACU, and reduce postoperative haemodynamic adverse events in a clinical setting in the absence of neuromuscular monitoring. This may enhance the patients’ recovery in the operating room and PACU while improving the postoperative condition of patients and may enhance the ERAS protocol.

## Abbreviations

ERAS, enhanced recovery after surgery; ASA, American Society of Anaesthesiologists; PACU, post-anaesthesia care unit; PONV, postoperative nausea and vomiting

## Additional file

Additional file 1: Available data. (XLSX 35 kb)
